# Anticoagulantes Orais Diretos versus Antagonistas da Vitamina K para Trombo Ventricular Esquerdo: Uma Metanálise com Análise Sequencial de Ensaios

**DOI:** 10.36660/abc.20230738

**Published:** 2024-07-10

**Authors:** Eric Pasqualotto, Douglas Mesadri Gewehr, Rafael Oliva Morgado Ferreira, Matheus Pedrotti Chavez, Caroliny Hellen Silva, Sara Almeida Cruz, Jhonny Limachi-Choque, Amanda Park, Mário Sérgio Soares de Azeredo Coutinho, Luiz Fernando Kubrusly

**Affiliations:** 1 Universidade Federal de Santa Catarina Florianópolis SC Brasil Universidade Federal de Santa Catarina, Florianópolis, SC – Brasil; 2 Faculdade Evangélica Mackenzie do Paraná Curitiba PR Brasil Faculdade Evangélica Mackenzie do Paraná, Curitiba, PR – Brasil; 3 Universidade Federal do Rio Grande do Norte Natal RN Brasil Universidade Federal do Rio Grande do Norte, Natal, RN – Brasil; 4 Immanuel Kant Baltic Federal University Institute of Medicine Kaliningrad Rússia Immanuel Kant Baltic Federal University Institute of Medicine, Kaliningrad – Rússia; 5 Universidad Mayor de San Simón Centro Universitario de Medicina Tropical Cochabamba Bolívia Universidad Mayor de San Simón - Centro Universitario de Medicina Tropical (CUMETROP), Cochabamba – Bolívia; 6 Centro Universitário Lusíada Faculdade de Ciências Médicas de Santos Santos SP Brasil Centro Universitário Lusíada - Faculdade de Ciências Médicas de Santos, Santos, SP – Brasil

**Keywords:** Varfarina, Inibidores do Fator Xa, Trombose

## Abstract

**Fundamento:**

Os antagonistas da vitamina K (AVKs) são o tratamento de primeira linha recomendado para trombo ventricular esquerdo (TVE); entretanto, os anticoagulantes orais diretos (AODs) têm sido considerados uma terapia alternativa.

**Objetivos:**

Avaliar a eficácia e a segurança dos AODs em comparação com a terapia com AVKs em pacientes com TVE.

**Métodos:**

PubMed, Embase e Cochrane foram sistematicamente pesquisados em busca de ensaios clínicos randomizados ou estudos de coorte que comparassem AODs versus AVKs para TVE. As razões de risco (RR) foram calculadas para desfechos binários, com intervalos de confiança (IC) de 95%. A significância estatística foi definida como valor de p < 0,05.

**Resultados:**

Foram incluídos um total de 4 ensaios clínicos randomizados e 29 estudos de coorte, com 4.450 pacientes designados para AODs ou AVKs. Não houve diferença significativa entre os grupos para acidente vascular cerebral ou eventos embólicos sistêmicos (AVC/EES) (RR 0,84; IC 95% 0,65 a 1,07; p = 0,157), acidente vascular cerebral (RR 0,73; IC 95% 0,48 a 1,11; p = 0,140), eventos embólicos sistêmicos (EES) (RR 0,69; IC 95% 0,40 a 1,17; p = 0,166), resolução do trombo (RR 1,05; IC 95% 0,99 a 1,11; p = 0,077), qualquer sangramento (RR 0,78; IC 95% 0,60 a 1,00; p = 0,054), sangramento clinicamente relevante (RR 0,69; IC 95% 0,46 a 1,03; p = 0,066), sangramento menor (RR 0,73; IC 95% 0,43 a 1,23; p = 0,234), sangramento maior (RR 0,87; IC 95% 0,42 a 1,80; p = 0,705) e mortalidade por todas as causas (RR 1,05; IC 95% 0,79 a 1,39; p = 0,752). Em comparação com AVKs, a rivaroxabana reduziu significativamente AVC/EES (RR 0,35; IC 95% 0,16 a 0,91; p = 0,029) e EES (RR 0,39; IC 95% 0,16 a 0,95; p = 0,037).

**Conclusões:**

Os AODs tiveram uma taxa semelhante de eventos tromboembólicos e hemorrágicos, bem como de resolução do trombo, em comparação com os AVKs no tratamento de TVE. A terapia com rivaroxabana teve uma redução significativa nos eventos tromboembólicos, em comparação com os AVKs.

## Graphical abstract

**Figura Central:**
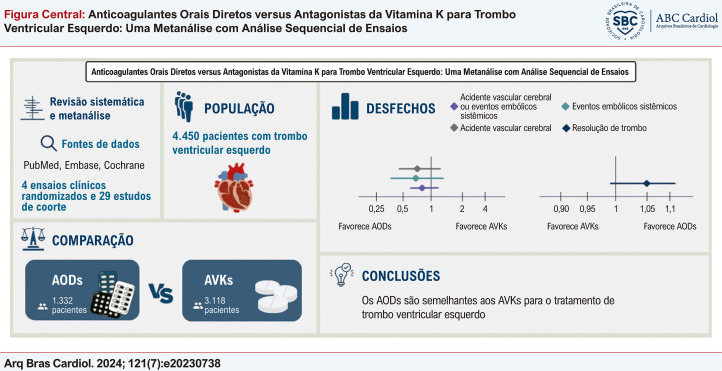
: Anticoagulantes Orais Diretos versus Antagonistas da Vitamina K para Trombo Ventricular Esquerdo: Uma Metanálise com Análise Sequencial de Ensaios

## Introdução

O trombo ventricular esquerdo (TVE) frequentemente ocorre como uma complicação de infarto agudo do miocárdio (IAM), cardiomiopatia não isquêmica ou disfunção cardíaca grave.^1^ Nos Estados Unidos, infartos do miocárdio ocorrem a uma taxa de 1 milhão por ano, e entre 4% e 39% desses pacientes podem desenvolver TVE, apresentando alta demanda por cuidados médicos.^2-4^ Apesar dos avanços na medicina cardiovascular, o tratamento de TVE, muitas vezes, permanece desafiador, devido a recomendações limitadas das diretrizes.^5^

Os antagonistas da vitamina K (AVKs) foram estabelecidos como prevenção e tratamento de TVE.^6^ O uso de AVKs está associado à necessidade de monitoramento frequente da razão normalizada internacional (RNI) e de vigilância para interações medicamentosas ou alimentares.^7^ A falha em manter a RNI na zona terapêutica está associada ao aumento da incidência de trombos.^7^ Nesse sentido, os anticoagulantes orais diretos (AODs) têm demonstrado eficácia semelhante aos AVKs, ao mesmo tempo em que apresentam menos complexidades de tratamento, levando ao aumento da sua utilização, apesar da ausência de orientação definitiva quanto à sua segurança como opção para pacientes com TVE.^8^

Metanálises anteriores, ensaios controlados randomizados (ECRs) e estudos retrospectivos comparando AODs com AVKs para o tratamento de TVE apresentam dados que apoiam o uso de AODs; no entanto, nem todos são consistentes, uma vez que foram observados resultados diferentes em relação aos eventos tromboembólicos e hemorrágicos.^9-11^ Assim, o regime de anticoagulação ideal para pacientes com TVE permanece desconhecido. Portanto, nosso objetivo foi realizar uma revisão sistemática e metanálise de ECRs e estudos observacionais, juntamente com uma análise sequencial de ensaios (TSA, do inglês *trial sequential analysis*) para comparar a eficácia e segurança de AODs versus AVKs em pacientes com TVE.

## Métodos

A presente revisão sistemática e metanálise seguiram as recomendações das diretrizes Preferred Reporting Items for Systematic Reviews and Meta-Analysis (PRISMA).^12^ O protocolo do estudo foi registrado no International Prospective Register of Systematic Reviews (PROSPERO) sob número de registro CRD42023409287.

### Estratégia de pesquisa e extração de dados

Os bancos de dados PubMed, Embase e Cochrane Library foram pesquisados sistematicamente desde o início até março de 2023, com a seguinte estratégia de pesquisa em inglês: (“left ventricular thrombus” OU “left ventricular thrombi” OU LVT OU LVTs) E (DOAC OU NOAC OU “direct anticoagulant” OU “direct oral anticoagulants” OU “direct oral anticoagulant” OU “oral anticoagulation” OU “new oral anticoagulant” OU rivaroxaban OU apixaban OU edoxaban OU dabigatran) E (“vitamin K antagonist” OU “vitamin K antagonists” OU VKA OU VKAs OU warfarin OU varfarin). Visando incluir estudos adicionais, foram analisadas referências de revisões sistemáticas e estudos incluídos para verificar a possibilidade de quaisquer outros estudos elegíveis. As características basais e os dados dos desfechos foram extraídos independentemente por dois autores (E.P. e R.O.M.F.). As divergências foram resolvidas por consenso com o autor sênior (E.P., R.O.M.F. e L.F.K.).

### Critérios de eligibilidade

Foram incluídos estudos que atenderam aos seguintes critérios: (1) ECRs ou estudos de coorte; (2) comparação de AODs com AVKs; (3) inscrição de pacientes com TVE; e (4) relato de pelo menos um parâmetro de interesse. Excluímos (1) populações sobrepostas; e (2) estudos que não eram ECRs ou estudos de coorte.

### Desfechos e análise de subgrupos

Os desfechos de interesse foram: (1) acidente vascular cerebral ou eventos embólicos sistêmicos (AVC/EES), (2) acidente vascular cerebral (AVC), (3) eventos embólicos sistêmicos (EES); (4) resolução do trombo; (5) qualquer sangramento; (6) sangramento clinicamente relevante; (7) sangramento menor; (8) sangramento maior; e (9) mortalidade por todas as causas.

A definição dos desfechos foi de acordo com os critérios estabelecidos nos estudos incluídos na presente revisão sistemática e metanálise. Qualquer sangramento incluiu todo sangramento. O sangramento clinicamente relevante incluiu sangramento não maior clinicamente relevante, sangramento menor e sangramento maior. Os ataques isquêmicos transitórios não foram considerados para análise do desfecho de AVC ou do desfecho composto de AVC ou EES.

As análises de subgrupos foram realizadas de acordo com: (1) tratamento com apixabana versus AVKs, (2) tratamento com rivaroxabana versus AVKs, (3) apenas ECRs, (4) pacientes com TVE pós-IAM e (5) exclusão de resumos de conferências.

### Avaliação de risco de viés

Os ECRs foram avaliados com a ferramenta da Cochrane Collaboration para avaliação de risco de viés em ensaios randomizados (RoB-2), com 5 domínios: seleção, desempenho, detecção, atrito e relato.^13^ O risco de viés de estudos de intervenção não randomizados (ROBINS-I, do inglês *Risk of Bias Summary for Non-randomized Studies*) foi utilizado para avaliar os estudos de coorte, com 7 domínios: confusão, seleção de participantes, classificação de intervenções, desvios das intervenções pretendidas, falta de dados, mensuração dos desfechos e resultado relatado.^14^ Dois autores independentes (E.P. e R.O.M.F.) realizaram a avaliação da qualidade. As divergências foram resolvidas por consenso com o autor sênior (E.P., R.O.M.F. e L.F.K.).

### Avaliação de qualidade

A qualidade geral da evidência foi analisada de acordo com as diretrizes de Classificação de Recomendações, Avaliação, Desenvolvimento e Análises (GRADE, do inglês *Grading of Recommendation, Assessment, Development and Evaluations*).^15^ Os desfechos foram classificados como evidência de qualidade muito baixa, baixa, moderada ou alta com base na presença de risco de viés, inconsistência de resultados, imprecisão, viés de publicação e magnitude dos efeitos do tratamento.

### Avaliação do risco de viés entre estudos

O potencial viés de publicação foi julgado para o desfecho de AVC/EES por inspeção visual de gráficos de funil com contorno aprimorado e avaliado pela assimetria de regressão de Egger e pelo teste de correlação de postos de Begg.^16,17^

### Análise estatística

Os efeitos do tratamento para desfechos binários foram comparados usando razões de risco (RR), com intervalos de confiança (IC) de 95%. A significância estatística foi definida como valor de p < 0,05. A heterogeneidade foi avaliada com o teste Q de Cochran e estatística I^2^; p < 0,10 e I^2^ > 25% foram considerados significativos para a heterogeneidade.^18^ O modelo de efeitos aleatórios do estimador de máxima verossimilhança restrita (REML, do inglês *restricted maximum-likelihood estimator*) foi usado para todos os desfechos.^19^ O software estatístico R, versão 4.2.1 (R Foundation for Statistical Computing) foi utilizado para a análise estatística.

### Análise de sensibilidade

A análise *leave-one-out* (exclusão de um estudo por vez) foi utilizada para identificar estudos influentes e seus efeitos nas estimativas agrupadas. Este procedimento foi realizado removendo dados de um estudo e reanalisando os dados restantes. Quando os valores de p do tamanho do efeito agrupado mudaram de significativo para não significativo ou vice-versa, a dominância do estudo foi atribuída.

### Análise sequencial de ensaios

Foi realizada TSA nos ECRs incluídos para avaliar se a evidência cumulativa tinha poder estatístico suficiente nos desfechos de resolução de trombos, AVC e sangramento clinicamente relevante. Nosso plano estatístico envolveu testes bilaterais com um erro tipo I de 5% e um erro tipo II de 20%. Os limites de monitoramento sequencial de teste (TSMBs, do inglês *trial sequential monitoring boundaries*) e convencionais foram gerados para os grupos AODs e VKAs. Uma correção de heterogeneidade foi aplicada na TSA utilizando o modelo de efeitos aleatórios com IC de 95%. Foi gerada uma curva de escore z para avaliar a confiança e adequação das evidências. O ajuste dos limiares para o escore z foi baseado na função de consumo alfa de O’Brien–Fleming. Além disso, foi realizada uma análise para determinar o número necessário de pacientes para aceitar ou rejeitar a intervenção. Utilizamos o programa TSA versão 0.9.5.10 beta (Copenhagen Trial Unit, Centre for Clinical Intervention Research, Rigshospitalet, Copenhagen, Dinamarca).^20^

## Resultados

### Seleção e características dos estudos

**Figura 1:**
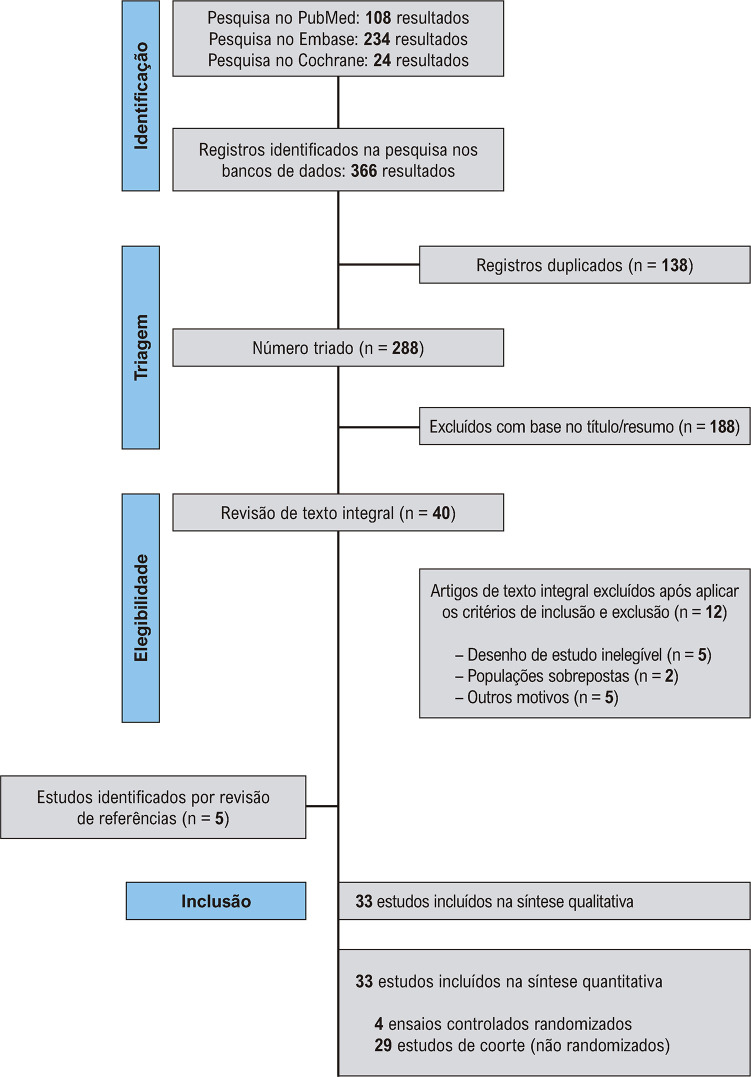
– Fluxograma PRISMA de triagem e seleção dos estudos.

Conforme ilustrado na Figura 1, a busca inicial rendeu 366 resultados. Após a remoção dos registros duplicados e dos estudos inelegíveis por título e resumo, restaram 40 estudos para revisão completa conforme critérios de inclusão. Além disso, foram identificados 5 estudos através do método de revisão de referências (*backward snowballing*). Destes, 4 ECRs e 29 estudos de coorte foram incluídos nesta revisão sistemática e metanálise, compreendendo 4.450 pacientes.^8,11,21-51^ Um total de 1.332 (29,9%) pacientes receberam AODs, enquanto 3.118 (70,3%) receberam AVKs. O período de acompanhamento variou de 3 meses a 3 anos. A média de idade variou de 49,6 a 69 anos. As características dos estudos e dos pacientes estão resumidas na Tabela 1 e no Material Suplementar 1, Tabelas S1 e S2.

**Tabela 1:** – Características de linha de base dos estudos incluídos

Estudo	Desenho do estudo	Número de pacientes, n			Acompanhamento, mediana ou média	Idade, anos, média (DP) ou mediana (IIQ)			Sexo, n		AODs
		AODs	AVKs	Total		AODs	AVKs	Total	Feminino	Masculino	
Al-abcha 2021*	Coorte retrospectivo	48	146	194	ND	ND	ND	61,6 (13,3)	46	148	ND
Albabtain 2021†	Coorte retrospectivo	28	35	63	ND	58,25 (17,73)	59 (15,62)	ND	5	58	Rivaroxabana
Alcalai 2021†	ECR	17	15	32	3 meses	55,5 (12,9)	58,8 (10,2)	57,1 (11,7)	4	28	Apixabana
Aldaas 2022†	Coorte retrospectivo	76	146	222	ND	ND	ND	ND	ND	ND	ND
Ali 2020*	Coorte retrospectivo	32	60	92	12 meses	59,2 (11,9)	58,0 (16,3)	59 (14)	17	75	Rivaroxabana, apixabana, dabigatrana
Alizadeh 2019†	Coorte prospectivo	38	60	98	1,8 anos	ND	ND	ND	ND	ND	Rivaroxabana, apixabana, edoxabana
Bass 2019†	Coorte retrospectivo	180	769	949	3 meses	63,4 (16,7)	61,6 (15,3)	ND	279	670	Rivaroxabana, apixabana, dabigatrana
Byrne 2022*	Coorte retrospectivo	16	41	57	12 meses	63 (58-67)	60 (50-70)	60 (53-69)	13	44	ND
Cochran 2021†	Coorte retrospectivo	14	59	73	13 meses	51,5 (39-73)	62 (34-84)	ND	17	56	Rivaroxabana, apixabana, dabigatrana, edoxabana
Conant 2022†	Coorte retrospectivo	29	135	164	ND	ND	ND	ND	ND	ND	ND
Daher 2020†	Coorte retrospectivo	17	42	59	ND	ND	ND	62 (14)	10	49	Rivaroxabana, apixabana, dabigatrana
Durrer-Ariyakuddy 2019*	Coorte	20	33	53	20 meses	ND	ND	63	14	39	ND
Gama 2019†	Coorte retrospectivo	12	52	64	ND	ND	ND	69 (12)	13	51	ND
Guddeti 2020†	Coorte retrospectivo	19	80	99	10,4 meses	60,7 (13,1)	61,3 (12,2)	61 (12,3)	29	70	Rivaroxabana, apixabana, dabigatrana
Haniff 2021*	ECR	14	13	27	3 meses	55,36 (11,04)	55,00 (11,42)	55,19 (11,01)	2	25	Apixabana
Harb 2022*	Coorte retrospectivo	22	59	81	6 meses	ND	ND	ND	ND	ND	ND
Iqbal 2020†	Coorte retrospectivo	22	62	84	3,0 anos	62 (13)	62 (14)	62 (14)	9	75	Rivaroxabana, apixabana, dabigatrana
Iskaros 2021†	Coorte retrospectivo	32	45	77	3 meses	62 (55–74)	63 (55–73)	ND	8	69	Rivaroxabana, apixabana, dabigatrana
Isom 2020*	Coorte retrospectivo	32	60	92	12 meses	ND	ND	59 (14)	ND	ND	ND
Jaidka 2018*	Coorte retrospectivo	12	37	49	6 meses	57,2 (9,3)	61,3 (12,1)	ND	12	37	ND
Jones 2020†	Coorte prospectivo	41	60	101	2,2 anos	58,73 (14,2)	60,81 (14,3)	ND	17	84	Rivaroxabana, apixabana, edoxabana
Mihm 2021*	Coorte retrospectivo	33	75	108	6 meses	63,3 (14,4)	60,3 (13,9)	ND	31	77	Rivaroxabana, apixabana
Minciunescu 2020*	Coorte retrospectivo	57	140	197	ND	60,4 (15,9)	59,5 (13,9)	ND	45	152	ND
No-LVT Trial 2021*	ECR	39	40	79	6 meses	ND	ND	49,6 (12,5)	34	45	Rivaroxabana
Robinson 2018*	Coorte retrospectivo	35	40	75	12 meses	ND	ND	ND	ND	ND	Rivaroxabana, apixabana, dabigatrana
Robinson 2020†	Coorte retrospectivo	121	236	357	351 dias	58,1 (14,9)	58,2 (15,1)	ND	93	264	Rivaroxabana, apixabana, dabigatrana
Seiler 2022*	Coorte retrospectivo	48	53	101	12 meses	ND	ND	63,3 (13,2)	18	83	ND
Willeford 2021†	Coorte retrospectivo	22	129	151	12 meses	54 (48-64)	56 (49-65,5)	56 (49-65)	30	121	Rivaroxabana, apixabana
Xu 2021†	Coorte retrospectivo	25	62	87	2,37 anos	59,4 (11,5)	61,9 (12,2)	61,5 (12,7)	21	66	Rivaroxabana, dabigatrana
Youssef 2023†	ECR	25	25	50	6 meses	52 (8,2)	53 (7,9)	ND	ND	ND	Apixabana
Yunis 2020†	Coorte retrospectivo	64	200	264	24 meses	ND	ND	ND	ND	ND	ND
Zhang 2021†	Coorte retrospectivo	33	31	64	25,0 meses	60,3 (14,7)	61,3 (9,0)	ND	17	47	Rivaroxabana
Zhang 2022†	Coorte retrospectivo	109	78	187	17,0 meses	64,5 (54,2–70,8)	63,0 (54,5–71,0)	ND	36	151	Rivaroxabana

*O nível de significância dos resultados não foi apresentado. †A significância estatística foi definida como valor de p < 0,05. AODs: anticoagulantes orais diretos; AVKs: antagonistas da vitamina K; DP: desvio padrão; ECR: ensaio controlado randomizado; IIQ: intervalo interquartil; ND: não disponível.

### Análise agrupada de todos os estudos

**Figura 2:**
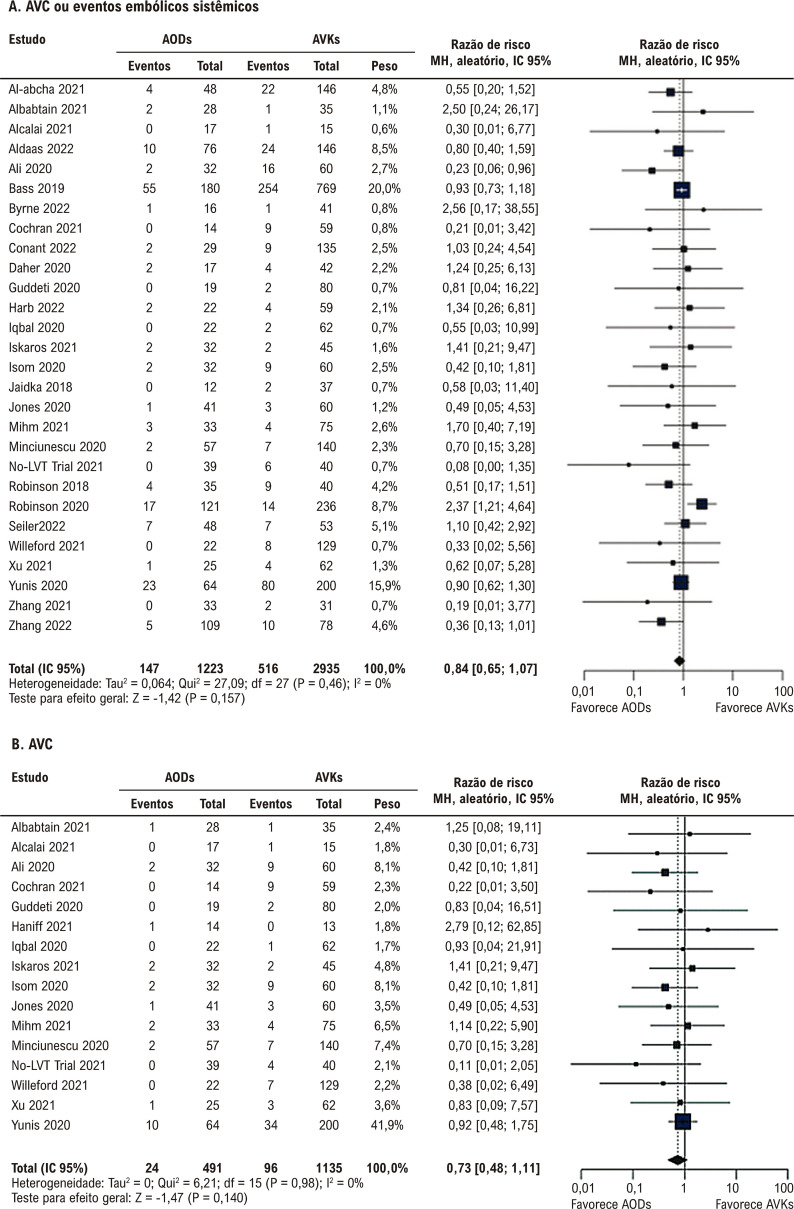
– Anticoagulantes orais diretos versus antagonistas da vitamina K. A) AVC ou eventos embólicos sistêmicos. B) AVC. AODs: anticoagulantes orais diretos; AVC: acidente vascular cerebral; AVKs: antagonistas da vitamina K; df: graus de liberdade; IC: intervalo de confiança; MH: Mantel-Haenszel.

Não houve diferença significativa entre a terapia com AODs e AVKs em relação a AVC/EES, AVC, EES e resolução de trombos (Figuras 2 e 3). Não houve diferença significativa entre os grupos em relação a qualquer sangramento, sangramento clinicamente relevante, sangramento menor, sangramento maior e mortalidade por todas as causas (Figuras 4 e 5).

**Figura 3:**
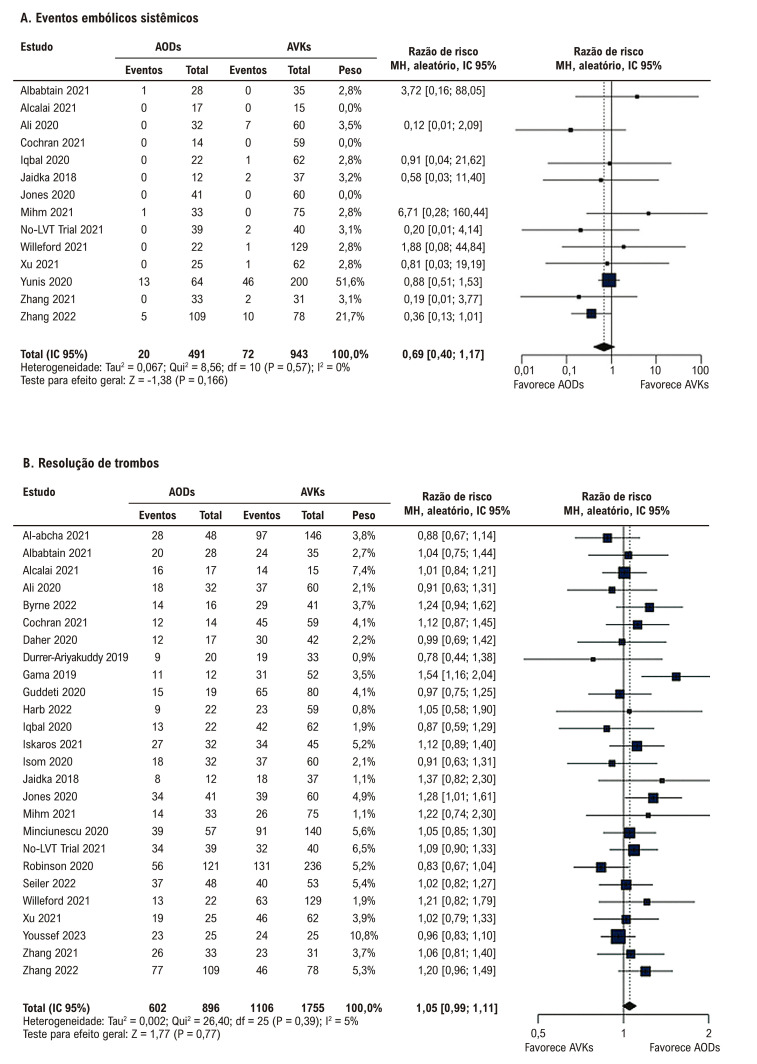
– Anticoagulantes orais diretos versus antagonistas da vitamina K. A) Eventos embólicos sistêmicos. B) Resolução de trombos. AODs: anticoagulantes orais diretos; AVKs: antagonistas da vitamina K; df: graus de liberdade; IC: intervalo de confiança; MH: Mantel-Haenszel.

**Figura 4:**
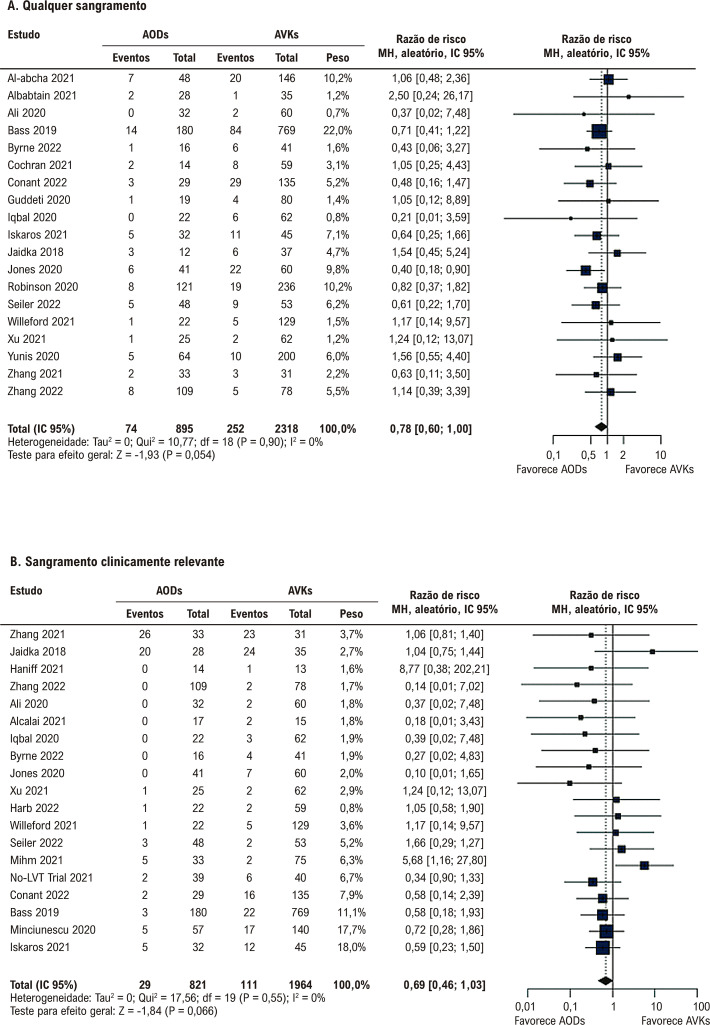
– Anticoagulantes orais diretos versus antagonistas da vitamina K. A) Qualquer sangramento. B) Sangramento clinicamente relevante. AODs: anticoagulantes orais diretos; AVKs: antagonistas da vitamina K; df: graus de liberdade; IC: intervalo de confiança; MH: Mantel-Haenszel.

**Figura 5:**
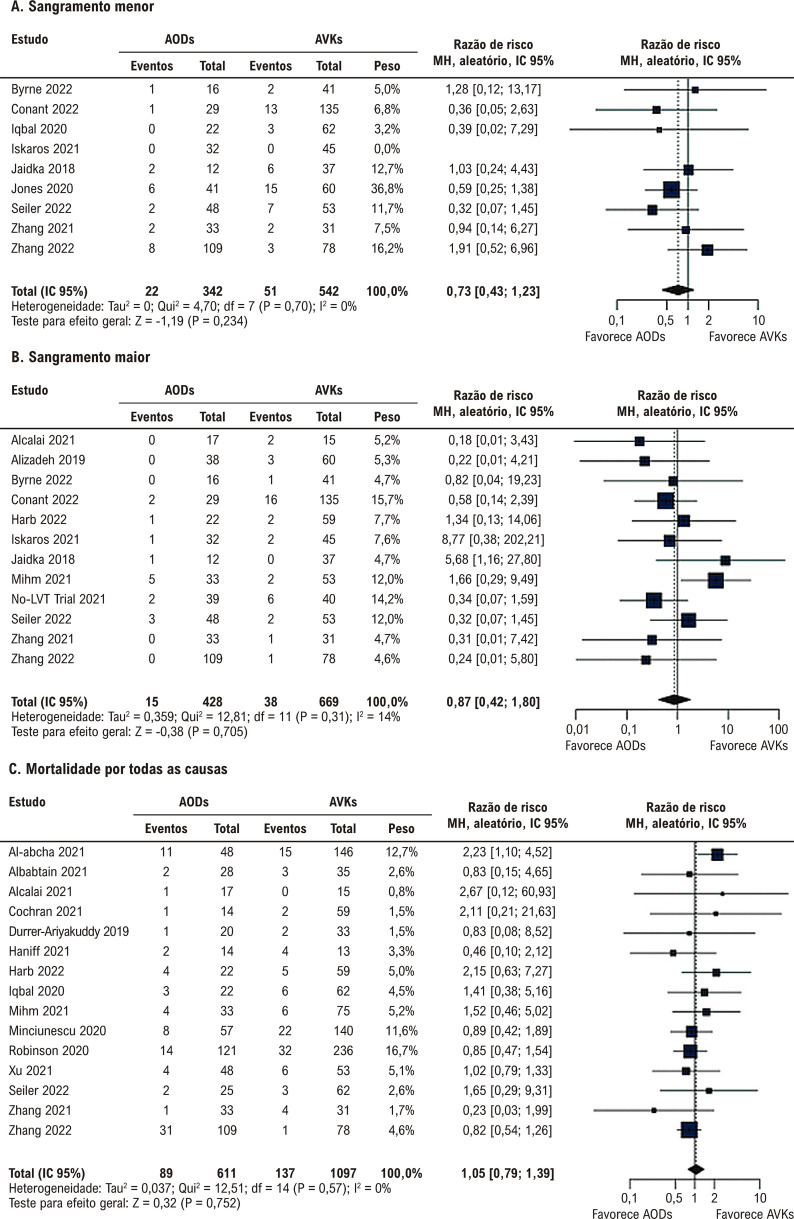
– Anticoagulantes orais diretos versus antagonistas da vitamina K. A) Sangramento menor. B) Sangramento maior. C) Mortalidade por todas as causas. AODs: anticoagulantes orais diretos; AVKs: antagonistas da vitamina K; df: graus de liberdade; IC: intervalo de confiança; MH: Mantel-Haenszel.

### Análise de subgrupos

Na análise de subgrupo de pacientes tratados com rivaroxabana,^21,22,47,48^ os AVC/EES e os EES foram significativamente reduzidos no grupo tratado com rivaroxabana. Não houve diferença significativa entre os grupos em relação a AVC, resolução de trombos, qualquer sangramento, sangramento clinicamente relevante, sangramento menor, sangramento maior e mortalidade por todas as causas. As análises agrupadas estão detalhadas no Material Suplementar 1, Figuras S1 e S2.

Na análise de subgrupo de pacientes tratados com apixabana,^8,49,51^ não houve diferença significativa entre os grupos em relação a AVC, resolução de trombos, sangramento clinicamente relevante e mortalidade por todas as causas. As análises agrupadas estão detalhadas no Material Suplementar 1, Figura S3.

Somente na análise de subgrupo dos ECRs,^8,22,49,51^ não houve diferença significativa entre os grupos em relação a AVC/EES, AVC, resolução de trombos, sangramento clinicamente relevante, sangramento maior e mortalidade por todas as causas. As análises agrupadas estão detalhadas no Material Suplementar 1, Figura S4.

Na análise de subgrupo incluindo pacientes com TVE após IAM,^8,26,28,39,40,47,51^ não houve diferença significativa entre os grupos em relação a AVC/EES, AVC, EES, resolução de trombos, qualquer sangramento, sangramento clinicamente relevante, sangramento leve, sangramento grave ou mortalidade por todas as causas. As análises agrupadas estão detalhadas no Material Suplementar 1, Figuras S5 e S6.

Na análise de subgrupo, excluindo resumos de conferências,^8,11,21,27,29,31,34,36,37,40,44,45,47,48,50,51^ qualquer sangramento foi significativamente reduzido no grupo tratado com AODs. No entanto, não houve diferença significativa entre os grupos em relação a AVC/EES, AVC, EES, resolução de trombos, sangramento clinicamente relevante, sangramento menor, sangramento maior ou mortalidade por todas as causas. As análises agrupadas estão detalhadas no Material Suplementar 1, Figuras S7, S8 e S9.

### Análise de sensibilidade

Realizamos uma análise de sensibilidade de exclusão para todos os desfechos. Houve uma diminuição significativa nos EES a favor da terapia com AODs, excluindo Robinson et al.^11^ Houve uma diferença significativa a favor da terapia com AODs em relação a qualquer sangramento, excluindo Al-abcha et al., Albabtain et al., Jaidka et al., Yunis et al., ou Zhang et al.^21,23,39,46,48^ Houve uma diferença significativa no sangramento clinicamente relevante em favor da terapia com AODs, excluindo Jaidka et al., Seiler et al. ou Mihm et al.^39,43,50^ Houve uma diferença significativa na resolução de trombos em favor da terapia com AODs, excluindo Robinson et al.^11^ Os gráficos de análise de sensibilidade de exclusão estão detalhados no Material Suplementar 1, Figuras S10 a S18.

### Avaliação de qualidade e evidências

Avaliações individuais dos ECR, de acordo com a ferramenta RoB-2, são ilustradas no Material Suplementar 1, Figura S19. No geral, todos os ECRs levantaram algumas preocupações devido a desvios das intervenções pretendidas,^8,49,51^ e um ECR levantou algumas preocupações devido à seleção dos resultados relatados.^49^

Não foi detectado viés de publicação significativo para o desfecho de AVC/EES pelo teste de Egger (p = 0,702) ou teste de Begg (p = 0,327). O gráfico de funil do desfecho de AVC/EES está disponível no Material Suplementar 1, Figura S21.

A avaliação crítica dos estudos de coorte está detalhada no Material Suplementar 1, Figura S20. Quatro estudos de coorte apresentaram baixo risco de viés,^29,40,47,48^ enquanto 10 estudos de coorte apresentaram risco moderado de viés, devido a vieses na seleção dos participantes.^11,21,27,31,34,36,37,44,45,50^ Um ECR e 15 estudos de coorte não forneceram informações suficientes para avaliar o risco de viés.^22-26,28,30,32,33,35,38,39,41-43,46^

De acordo com a avaliação GRADE, foi atribuída qualidade muito baixa a todos os desfechos, principalmente devido à inclusão de resumos e múltiplos estudos sem informações sobre risco de viés. O Material Suplementar 2 relata a avaliação GRADE completa e o resumo dos achados.

### Análise sequencial de ensaios

**Figura 6:**
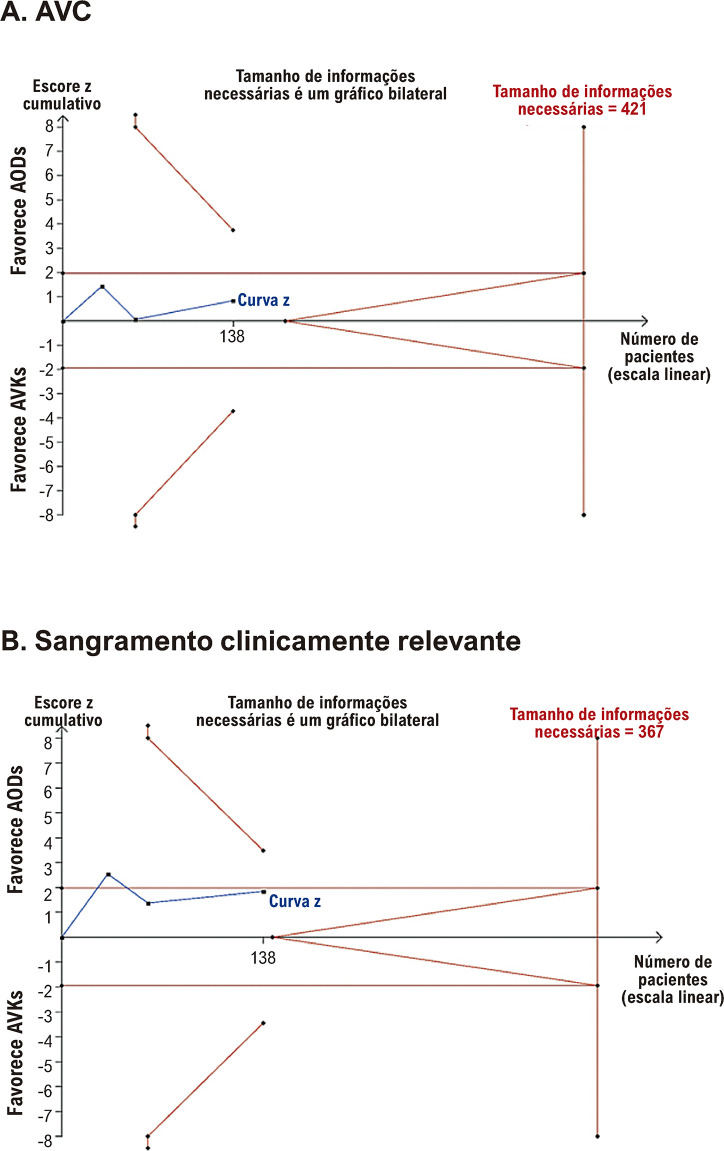
– Análise sequencial de ensaios para (A) acidente vascular cerebral e (B) sangramento clinicamente relevante. AODs: anticoagulantes orais diretos; AVC: acidente vascular cerebral; AVKs: antagonistas da vitamina K.

A curva z cumulativa para AVC e sangramento clinicamente relevante não ultrapassou os limites convencionais e de monitoramento e não atingiu o tamanho de informações necessárias (RIS, do inglês *required information size*). Neste caso, não podemos concluir se os resultados neutros resultam de falta de poder ou se é pouco provável que a intervenção tenha um impacto significativo. Para resolução de trombos, o último ponto da curva z encontra-se dentro dos limites de futilidade, indicando que dificilmente alcançará significância estatística, mesmo se procedermos com inclusão de ensaios randomizados de pacientes até o RIS de 367. Os gráficos sequenciais dos ensaios estão detalhados nas Figura 6 e 7.

**Figura 7:**
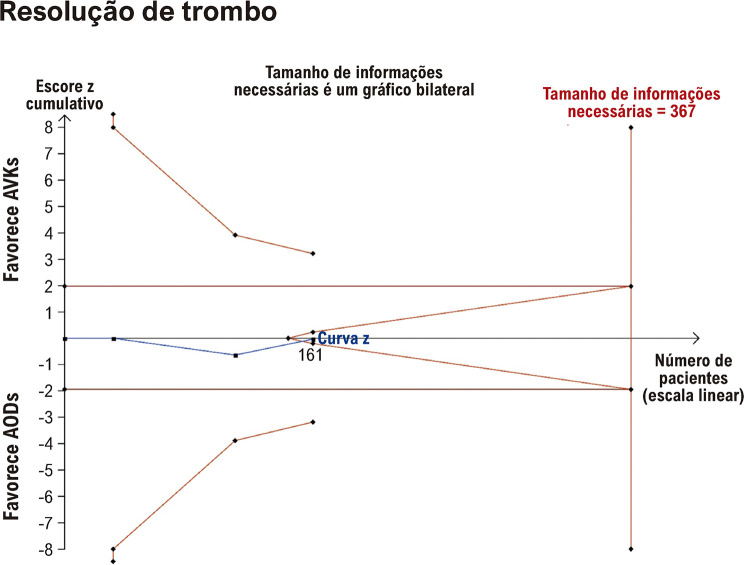
– Análise sequencial de ensaios para resolução de trombos. AODs: anticoagulantes orais diretos; AVKs: antagonistas da vitamina K.

## Discussão

Na presente revisão sistemática e metanálise composta por 33 estudos e 4.450 pacientes, comparamos a eficácia de 2 tipos de anticoagulantes, AODs e AVKs, para o tratamento de TVE. Nossos achados foram: (I) a terapia com AODs foi equivalente aos AVKs para TVE na ocorrência de eventos tromboembólicos e resolução de trombos; (II) a rivaroxabana reduziu significativamente os eventos tromboembólicos; e (III) a ocorrência de complicações hemorrágicas foi semelhante entre os grupos.

TVE constitui um fator etiológico proeminente para AVC embólico subsequente a IAM e insuficiência cardíaca congestiva.^52,53^ Maniwa et al. revelaram que indivíduos com TVE podem apresentar EES com uma taxa de incidência de até 16,3%, que é 5 vezes maior do que aqueles sem TVE,^54^ e mais de 10% morrem dentro de 1 ano.^55^ As diretrizes europeias e estadunidenses recomendam terapia anticoagulante por 3 a 6 meses em pacientes com TVE.^6,56^ Os AVKs, principalmente a varfarina, são indicados como anticoagulantes orais de primeira linha para o tratamento de TVE.^9^ No entanto, o uso da varfarina apresenta desvantagens, incluindo interações com medicamentos e alimentos, variabilidade nas respostas individuais, exigência de monitoramento frequente e necessidade de usar heparina não fracionada ou heparina de baixo peso molecular durante, pelo menos, os 3 dias iniciais devido a um atraso na inibição do fator II.^57^

Na última década, novos AODs foram aprovados para tratamento anticoagulante da fibrilação atrial (FA) não valvar e doenças tromboembólicas venosas.^58,59^ A ação anticoagulante dos AODs é baseada na inibição da trombina, com dabigatrana, ou fator Xa, com rivaroxabana, edoxabana e apixabana.^57,59^ Atualmente, está comprovado que os AODs são superiores à varfarina no tratamento e prevenção de eventos tromboembólicos em pacientes com FA não valvular.^60^ Para TVE, os AODs podem ser aplicáveis, pois o mecanismo fisiopatológico é semelhante ao da trombose relacionada à FA.^57^ No entanto, nenhuma diretriz formal atualizada recomendou o uso de AODs em pacientes com TVE. As diretrizes sobre IAMCSST da American College of Cardiology/American Heart Association de 2013 e as diretrizes sobre IAMCSST da Sociedade Europeia de Cardiologia de 2017 não fazem referência ao uso de AODs na anticoagulação para TVE.^6,56^ No entanto, a diretriz sobre AVC de 2021 da American Heart Association/American Stroke Association apresentou uma recomendação de classe IIb apoiando o uso de AODs para reduzir o risco de trombose recorrente em pacientes com AVC ou ataque isquêmico transitório e novos TVEs.^5,9^ Assim, os AODs têm sido usados como tratamento *off-label* para TVE, com orientações muito limitadas sobre seu uso.^57,61^

Em uma metanálise publicada como declaração científica pela American Heart Association, os AODs foram considerados uma alternativa razoável aos AVKs em pacientes com TVE.^9^ Essa abordagem de tratamento foi particularmente interessante para pacientes para os quais a manutenção de uma faixa terapêutica consistente da RNI se mostra um desafio ou para aqueles que não podem realizar monitoramento frequente da RNI.^9^ Em metanálises anteriores, Michael et al. mostraram taxas reduzidas de AVC em pacientes tratados com AODs em comparação com AVKs, com resolução de trombos e eventos hemorrágicos semelhantes.^62^ Da mesma maneira, Trongtorsak et al. demonstraram taxas semelhantes de eventos tromboembólicos sistêmicos, resolução de trombos e sangramento.^63^ Chen et al. e Kido et al. mostraram uma redução significativa no sangramento em pacientes tratados com AODs em comparação com AVKs, com taxas semelhantes de eventos tromboembólicos.^57,64^ Na presente metanálise, verificamos uma redução significativa em qualquer sangramento em favor dos AODs apenas na análise de subgrupo, excluindo resumos de conferências. Considerando que a exclusão de resumos de conferências reduz a possibilidade de viés na análise estatística, esse resultado favorece a interpretação da existência de benefício dos AODs para o tratamento de TVE em comparação aos AVKs.

Li et al. e Ferreira et al. não encontraram diferenças entre AODs e AVKs para eventos tromboembólicos ou sangramento.^61,65^ Assim, a nossa metanálise também não indicou diferenças estatísticas para os resultados de AVC, EES ou o resultado composto de AVC/EES. No entanto, até onde sabemos, esta é a primeira metanálise que realizou análises de subgrupos para apixabana e rivaroxabana, encontrando um benefício da rivaroxabana na redução de eventos tromboembólicos, avaliado pela redução significativa de AVC/EES e EES quando comparado com AVKs.

Na TSA, a evidência sólida é alcançada quando o tamanho amostral de pacientes excede o necessário para se chegar a uma conclusão definitiva ou quando as curvas z cruzam os TSMBs antes de atingir a contagem essencial de pacientes para obter evidências conclusivas. Pelo contrário, nos casos em que a curva z cruza os limiares estatísticos convencionais, mas não os TSMBs e a amostra de pacientes necessária para aceitar ou rejeitar a hipótese, o efeito significativo da metanálise pode resultar de testes repetitivos, em vez de efeitos subjacentes genuínos. Nos casos em que o número de participantes na metanálise excede a linha RIS, isso sugere que existem evidências suficientes para tirar conclusões confiáveis sobre o efeito da intervenção. Em nossa metanálise, a TSA não mostrou evidências suficientes do benefício dos AODs em relação aos AVKs no tratamento de TVE em relação à resolução de trombos, AVC e sangramento clinicamente relevante.

O presente estudo deve ser interpretado considerando suas limitações. Primeiro, a maioria dos estudos incluídos nesta metanálise eram estudos de coorte, e os tamanhos das amostras, especialmente nos ECR incluídos, eram pequenos. Segundo, as características dos pacientes, o tipo dos AODs, a definição de eventos clínicos e o acompanhamento mostraram variações entre os estudos incluídos, contribuindo para a heterogeneidade entre estudos. Terceiro, a inclusão de resumos de conferências representa uma fonte potencial de viés na análise, uma vez que os resumos muitas vezes não incluem informações sobre a população de pacientes e não é possível avaliar os riscos de viés devido à falta inerente de informações detalhadas sobre o desenho do estudo. Além disso, devido à informação limitada fornecida nos resumos da conferência, a sobreposição de populações é uma preocupação, embora nenhum resumo incluído na nossa metanálise tenha tido o mesmo número de pacientes que os estudos publicados na íntegra. Quarto, a avaliação GRADE exibiu uma qualidade de evidência muito baixa devido à heterogeneidade significativa entre estudos e um grave risco de viés. Quinto, as análises de subgrupos foram realizadas com um tamanho amostral pequeno. Sexto, as análises de subgrupos para diferentes AODs, exceto rivaroxabana e apixabana, não foram viáveis devido à falta de dados adequados. Portanto, a generalização dos resultados é limitada. São necessários ECRs maiores para confirmar a eficácia e segurança dos AODs em comparação aos AVKs para o tratamento de TVE.

## Conclusão

Os AODs tiveram uma taxa semelhante de eventos tromboembólicos e hemorrágicos, bem como de resolução de trombos, em comparação com os AVKs no tratamento de TVE. A terapia com rivaroxabana teve uma redução significativa nos eventos tromboembólicos, em comparação com os AVKs.
