# Supplementary material for: Anticoagulantes Orais Diretos versus Antagonistas da Vitamina K para Trombo Ventricular Esquerdo: Uma Metanálise com Análise Sequencial de Ensaios
Source: Arq Bras Cardiol. 2024 Jul 10;121(7):e20230738. [Article in Portuguese] doi: 10.36660/abc.20230738 (PMC12080627; doi:10.36660/abc.20230738)
Supplement: Translation

# Translation: Direct Oral Anticoagulants versus Vitamin K Antagonists for Left Ventricular Thrombus: A Meta-Analysis with Trial Sequential Analysis

**DOI:** 10.36660/abc.20230738i

**Authors:** Eric Pasqualotto, Douglas Mesadri Gewehr, Rafael Oliva Morgado Ferreira, Matheus Pedrotti Chavez, Caroliny Hellen Silva, Sara Almeida Cruz, Jhonny Limachi-Choque, Amanda Park, Mário Sérgio Soares de Azeredo Coutinho, Luiz Fernando Kubrusly

**Affiliations:** 1 Brazil Universidade Federal de Santa Catarina, Florianópolis, SC – Brazil; 2 Brazil Faculdade Evangélica Mackenzie do Paraná, Curitiba, PR – Brazil; 3 Brazil Universidade Federal do Rio Grande do Norte, Natal, RN – Brazil; 4 Russia Immanuel Kant Baltic Federal University Institute of Medicine, Kaliningrad – Russia; 5 Bolivia Universidad Mayor de San Simón - Centro Universitario de Medicina Tropical (CUMETROP), Cochabamba – Bolivia; 6 Brazil Centro Universitário Lusíada - Faculdade de Ciências Médicas de Santos, Santos, SP – Brazil

**Keywords:** Warfarin, Factor Xa Inhibitors, Thrombosis

## Abstract

**Background:**

Vitamin K antagonists (VKAs) are the recommended first-line treatment for left ventricular thrombus (LVT); however, direct oral anticoagulants (DOACs) have been considered an alternative therapy.

**Objectives:**

To evaluate the efficacy and safety of DOACs compared with VKAs therapy in patients with LVT.

**Methods:**

PubMed, Embase, and Cochrane were systematically searched for randomized clinical trials or cohort studies that compared DOACs versus VKAs for LVT. Risk ratios (RRs) were computed for binary endpoints, with 95% confidence intervals (95% CIs). Statistical significance was defined as p value < 0.05.

**Results:**

A total of 4 randomized clinical trials and 29 cohort studies were included, with 4,450 patients assigned to either DOACs or VKAs. There was no significant difference between groups for stroke or systemic embolic (SSE) events (RR 0.84; 95% CI 0.65 to 1.07; p = 0.157), stroke (RR 0.73; 95% CI 0.48 to 1.11; p = 0.140), systemic embolic (SE) events (RR 0.69; 95% CI 0.40 to 1.17; p = 0.166), thrombus resolution (RR 1.05; 95% CI 0.99 to 1.11; p = 0.077), any bleeding (RR 0.78; 95% CI 0.60 to 1.00; p = 0.054), clinically relevant bleeding (RR 0.69; 95% CI 0.46 to 1.03; p = 0.066), minor bleeding (RR 0.73; 95% CI 0.43 to 1.23; p = 0.234), major bleeding (RR 0.87; 95% CI 0.42 to 1.80; p = 0.705), and all-cause mortality (RR 1.05; 95% CI 0.79 to 1.39; p = 0.752). Compared with VKAs, rivaroxaban significantly reduced SSE events (RR 0.35; 95% CI 0.16 to 0.91; p = 0.029) and SE events (RR 0.39; 95% CI 0.16 to 0.95; p = 0.037).

**Conclusions:**

DOACs had a similar rate of thromboembolic and hemorrhagic events, as well as thrombus resolution, compared to VKAs in the treatment of LVTs. Rivaroxaban therapy had a significant reduction in thromboembolic events, compared to VKAs.

## Introduction

Left ventricular thrombus (LVT) commonly occurs as a complication of acute myocardial infarction (AMI), nonischemic cardiomyopathy, or severe cardiac dysfunction.^1^ In the United States, myocardial infarctions occur at a rate of 1 million per year, and 4% to 39% of these patients can develop LVT, presenting a high demand for medical care.^2-4^ Despite the advances in cardiovascular medicine, the treatment of LVT often remains challenging, due to limited guideline recommendations.^5^

Vitamin K antagonists (VKAs) have been established as the prevention and treatment of LVT.^6^ The use of VKAs is associated with the need for frequent international normalized ratio (INR) monitoring and vigilance for drug or food interactions.^7^ The failure to maintain the INR in the therapeutic zone is associated with an increase in the incidence of thrombus.^7^ In this sense, direct oral anticoagulants (DOACs) have demonstrated similar effectiveness to VKAs while presenting fewer treatment complexities, leading to their increased utilization, despite the absence of definitive guidance regarding their safety as an option for patients with LVT.^8^

Previous meta-analyses, randomized controlled trials (RCTs), and retrospective studies comparing DOACs with VKAs for the treatment of LVT present data that support the use of DOACs; however, not all are consistent since different results were observed regarding thromboembolic and hemorrhagic events.^9-11^ Thus, the optimal anticoagulation regimen for patients with LVT remains unknown. Therefore, we aimed to perform a systematic review and meta-analysis of RCTs and observational studies along with a trial sequential analysis (TSA) to compare the efficacy and safety of DOACs versus VKAs in patients with LVT.

## Methods

This systematic review and meta-analysis followed the recommendations of the Preferred Reporting Items for Systematic Reviews and Meta-Analysis (PRISMA) guidelines.^12^ The study protocol was registered in the International Prospective Register of Systematic Reviews (PROSPERO) with registration number CRD42023409287.

### Search strategy and data extraction

PubMed, Embase, and Cochrane Library databases were systematically searched from inception to March 2023, with the following search strategy: (“left ventricular thrombus” OR “left ventricular thrombi” OR LVT OR LVTs) AND (DOAC OR NOAC OR “direct anticoagulant” OR “direct oral anticoagulants” OR “direct oral anticoagulant” OR “oral anticoagulation” OR “new oral anticoagulant” OR rivaroxaban OR apixaban OR edoxaban OR dabigatran) AND (“vitamin K antagonist” OR “vitamin K antagonists” OR VKA OR VKAs OR warfarin OR varfarin). Aiming to include additional studies, references of systematic reviews and included studies were analyzed to verify the possibility of any other eligible studies. Baseline characteristics and outcome data were extracted independently by two authors (E.P. and R.O.M.F.). Disagreements were resolved by consensus with the senior author (E.P., R.O.M.F., and L.F.K.).

### Eligibility criteria

Studies that met the following criteria were included: (1) RCTs or cohort studies; (2) comparing DOACs with VKAs; (3) enrolling patients with LVT; and (4) reporting at least one endpoint of interest. We excluded (1) overlapping populations; and (2) non-RCTs or non-cohort studies.

### Endpoints and subgroup analysis

Outcomes of interest were: (1) stroke or systemic embolic (SSE) events, (2) stroke, (3) systemic embolic (SE) events; (4) thrombus resolution; (5) any bleeding; (6) clinically relevant bleeding; (7) minor bleeding; (8) major bleeding; and (9) all-cause mortality.

The definition of outcomes was in accordance with the criteria established in the studies included in this systematic review and meta-analysis. Any bleeding included all bleeding. Clinically relevant bleeding included clinically relevant non-major bleeding, minor bleeding, and major bleeding. Transient ischemic attacks were not considered for analysis of the stroke outcome or the composite outcome of stroke or systemic embolic events.

Subgroup analyses were performed according to: (1) treatment with apixaban versus VKAs, (2) treatment with rivaroxaban versus VKAs, (3) RCTs only, (4) patients with LVT post-AMI, and (5) excluding conference abstracts.

### Risk of bias assessment

RCTs were appraised with the Cochrane Collaboration’s tool for assessing risk of bias in randomized trials (RoB-2), with 5 domains: selection, performance, detection, attrition, and reporting.^13^ Risk Of Bias In Non-randomized Studies of Interventions (ROBINS-I) was used to evaluate the cohort studies, with 7 domains: confounding, selection of participants, classification of interventions, deviations from intended interventions, missing data, measurement of outcomes, and reported result.^14^ Two independent authors (E.P. and R.O.M.F.) conducted the quality assessment. Disagreements were resolved by consensus with the senior author (E.P., R.O.M.F, and L.F.K.).

### Quality assessment

The overall quality of evidence was analyzed according to the Grading of Recommendation, Assessment, Development and Evaluations (GRADE) guidelines.^15^ The outcomes were labeled as very low, low, moderate, or high-quality evidence based on the presence of risk of bias, inconsistency of results, imprecision, publication bias, and magnitude of treatment effects.

### Assessment of risk of bias across studies

Potential publication bias was judged for the SSE events outcome by visual inspection of contour-enhanced funnel plots and assessed by Egger’s regression asymmetry and Begg’s rank correlation test.^16,17^

### Statistical analysis

The treatment effects for binary endpoints were compared using risk ratios (RRs), with 95% confidence intervals (CIs). Statistical significance was defined as p value < 0.05. The heterogeneity was assessed with Cochran Q test and I^2^ statistics; p < 0.10 and I^2^ > 25% were considered significant for heterogeneity.^18^ Restricted maximum-likelihood estimator (REML) random-effects model was used for all endpoints.^19^ R statistical software, version 4.2.1 (R Foundation for Statistical Computing) was used for statistical analysis.

### Sensitivity analyses

Leave-one-out procedures were used to identify influential studies and their effect on the pooled estimates. This procedure was carried out by removing data from one study and reanalyzing the remaining data. When pooled effect size p values changed from significant to non-significant or vice-versa, study dominance was assigned.

### Trial sequential analysis

A TSA was conducted on the included RCTs to assess whether the cumulative evidence had sufficient statistical power in thrombus resolution, stroke, and clinically relevant bleeding outcomes. Our statistical plan involved two-sided testing with a type I error of 5% and a type II error of 20%. Both conventional and trial sequential monitoring boundaries (TSMBs) were generated for the DOACs and VKAs groups. A heterogeneity correction was applied in the TSA using the random effects model with 95% CIs. A z score curve was generated to assess the confidence and adequacy of evidence. The adjustment of the thresholds for the z score was based on the O’Brien–Fleming alpha spending function. Additionally, an analysis to determine the required number of patients to either accept or reject the intervention was performed. We used the TSA program version 0.9.5.10 beta (Copenhagen Trial Unit, Centre for Clinical Intervention Research, Rigshospitalet, Copenhagen, Denmark).^20^

## Results

### Study selection and characteristics

As illustrated in Figure 1, the initial search yielded 366 results. After the removal of duplicate records and ineligible studies by title and abstract, 40 studies remained for full review according to inclusion criteria. In addition, 5 studies were identified through backward snowballing. Of these, 4 RCTs and 29 cohort studies were included in this systematic review and meta-analysis, comprising 4,450 patients.^8,11,21-51^ A total of 1,332 (29.9%) patients received DOACs, while 3,118 (70.3%) received VKAs. The follow-up period ranged from 3 months to 3 years. The mean age ranged from 49.6 to 69 years. Study and patient characteristics are summarized in Table 1 and Supplementary Material 1, Tables S1 and S2.

**Figure 1:** – PRISMA flow diagram of study screening and selection.

**Tabela 1:** – Baseline characteristics of included studies

Study	Study design	Number of patients, n			Follow-up, median or mean	Age, years, mean (SD) or median (QR)			Sex, n		DOACs
		DOACs	VKAs	Total		DOACs	VKAs	Total	Female	Male	
Al-abcha 2021*	Retrospective cohort	48	146	194	NA	NA	NA	61.6 (13.3)	46	148	NA
Albabtain 2021†	Retrospective cohort	28	35	63	NA	58.25 (17.73)	59 (15.62)	NA	5	58	Rivaroxaban
Alcalai 2021†	RCT	17	15	32	3 months	55.5 (12.9)	58.8 (10.2)	57.1 (11.7)	4	28	Apixaban
Aldaas 2022†	Retrospective cohort	76	146	222	NA	NA	NA	NA	NA	NA	NA
Ali 2020*	Retrospective cohort	32	60	92	12 months	59.2 (11.9)	58.0 (16.3)	59 (14)	17	75	Rivaroxaban, apixaban, dabigatran
Alizadeh 2019†	Prospective cohort	38	60	98	1.8 years	NA	NA	NA	NA	NA	Rivaroxaban, apixaban, edoxaban
Bass 2019†	Retrospective cohort	180	769	949	3 months	63.4 (16.7)	61.6 (15.3)	NA	279	670	Rivaroxaban, apixaban, dabigatran
Byrne 2022*	Retrospective cohort	16	41	57	12 months	63 (58-67)	60 (50-70)	60 (53-69)	13	44	NA
Cochran 2021†	Retrospective cohort	14	59	73	13 months	51.5 (39-73)	62 (34-84)	NA	17	56	Rivaroxaban, apixaban, dabigatran, edoxaban
Conant 2022†	Retrospective cohort	29	135	164	NA	NA	NA	NA	NA	NA	NA
Daher 2020†	Retrospective cohort	17	42	59	NA	NA	NA	62 (14)	10	49	Rivaroxaban, apixaban, dabigatran
Durrer-Ariyakuddy 2019*	Cohort	20	33	53	20 months	NA	NA	63	14	39	NA
Gama 2019†	Retrospective cohort	12	52	64	NA	NA	NA	69 (12)	13	51	NA
Guddeti 2020†	Retrospective cohort	19	80	99	10.4 months	60.7 (13.1)	61.3 (12.2)	61 (12.3)	29	70	Rivaroxaban, apixaban, dabigatran
Haniff 2021*	RCT	14	13	27	3 months	55.36 (11.04)	55.00 (11.42)	55.19 (11.01)	2	25	Apixaban
Harb 2022*	Retrospective cohort	22	59	81	6 months	NA	NA	NA	NA	NA	NA
Iqbal 2020†	Retrospective cohort	22	62	84	3.0 years	62 (13)	62 (14)	62 (14)	9	75	Rivaroxaban, apixaban, dabigatran
Iskaros 2021†	Retrospective cohort	32	45	77	3 months	62 (55–74)	63 (55–73)	NA	8	69	Rivaroxaban, apixaban, dabigatran
Isom 2020*	Retrospective cohort	32	60	92	12 months	NA	NA	59 (14)	NA	NA	NA
Jaidka 2018*	Retrospective cohort	12	37	49	6 months	57.2 (9.3)	61.3 (12.1)	NA	12	37	NA
Jones 2020†	Prospective cohort	41	60	101	2.2 years	58.73 (14.2)	60.81 (14.3)	NA	17	84	Rivaroxaban, apixaban, edoxaban
Mihm 2021*	Retrospective cohort	33	75	108	6 months	63.3 (14.4)	60.3 (13.9)	NA	31	77	Rivaroxaban, apixaban
Minciunescu 2020*	Retrospective cohort	57	140	197	NA	60.4 (15.9)	59.5 (13.9)	NA	45	152	NA
No-LVT Trial 2021*	RCT	39	40	79	6 months	NA	NA	49.6 (12.5)	34	45	Rivaroxaban
Robinson 2018*	Retrospective cohort	35	40	75	12 months	NA	NA	NA	NA	NA	Rivaroxaban, apixaban, dabigatran
Robinson 2020†	Retrospective cohort	121	236	357	351 days	58.1 (14.9)	58.2 (15.1)	NA	93	264	Rivaroxaban, apixaban, dabigatran
Seiler 2022*	Retrospective cohort	48	53	101	12 months	NA	NA	63.3 (13.2)	18	83	NA
Willeford 2021†	Retrospective cohort	22	129	151	12 months	54 (48-64)	56 (49-65.5)	56 (49-65)	30	121	Rivaroxaban, apixaban
Xu 2021†	Retrospective cohort	25	62	87	2.37 years	59.4 (11.5)	61.9 (12.2)	61.5 (12.7)	21	66	Rivaroxaban, dabigatran
Youssef 2023†	RCT	25	25	50	6 months	52 (8.2)	53 (7.9)	NA	NA	NA	Apixaban
Yunis 2020†	Retrospective cohort	64	200	264	24 months	NA	NA	NA	NA	NA	NA
Zhang 2021†	Retrospective cohort	33	31	64	25.0 months	60.3 (14.7)	61.3 (9.0)	NA	17	47	Rivaroxaban
Zhang 2022†	Retrospective cohort	109	78	187	17.0 months	64.5 (54.2–70.8)	63.0 (54.5–71.0)	NA	36	151	Rivaroxaban

*The significance level of the results was not presented. †Statistical significance was defined as p value < 0.05. DOACs: direct oral anticoagulants; IQR: interquartile range; NA: not available; RCT: randomized controlled trial; SD: standard deviations; VKAs: vitamin K antagonists.

### Pooled analysis of all studies

There was no significant difference between DOAC and VKA therapy regarding SSE events, stroke, SE events, and thrombus resolution (Figures 2 and 3). There was no significant difference between groups regarding any bleeding, clinically relevant bleeding, minor bleeding, major bleeding, and all-cause mortality (Figures 4 and 5).

**Figure 2:** – Direct oral anticoagulants (DOACs) versus vitamin K antagonists (VKAs). A) Stroke or systemic embolic events. B) Stroke. CI: confidence interval; MH: Mantel-Haenszel.

**Figure 3:** – Direct oral anticoagulants (DOACs) versus vitamin K antagonists (VKAs). A) Systemic embolic events. B) Thrombus resolution. CI: confidence interval; MH: Mantel-Haenszel.

**Figure 4:** – Direct oral anticoagulants (DOACs) versus vitamin K antagonists (VKAs). A) Any bleeding. B) Clinically relevant bleeding. CI: confidence interval; MH: Mantel-Haenszel.

**Figure 5:** – Direct oral anticoagulants (DOACs) versus vitamin K antagonists (VKAs). A) Minor bleeding. B) Major bleeding. C) All-cause mortality. CI: confidence interval; MH: Mantel-Haenszel.

### Subgroup analysis

In the subgroup analysis of patients treated with rivaroxaban,^21,22,47,48^ SSE events and SE events were significantly reduced in the group treated with rivaroxaban. There was no significant difference between groups regarding stroke, thrombus resolution, any bleeding, clinically relevant bleeding, minor bleeding, major bleeding, and all-cause mortality. The pooled analyses are detailed in Supplementary Material 1, Figures S1 and S2.

In the subgroup analysis of patients treated with apixaban,^8,49,51^ there was no significant difference between groups regarding stroke, thrombus resolution, clinically relevant bleeding, and all-cause mortality. The pooled analyses are detailed in Supplementary Material 1, Figure S3.

In the subgroup analysis of RCTs only,^8,22,49,51^ there was no significant difference between groups regarding SSE events, stroke, thrombus resolution, clinically relevant bleeding, major bleeding, and all-cause mortality. The pooled analyses are detailed in Supplementary Material 1, Figure S4.

In the subgroup analysis including patients with LVT post-AMI,^8,26,28,39,40,47,51^ there was no significant difference between groups regarding SEE events, stroke, SE events, thrombus resolution, any bleeding, clinically relevant bleeding, minor bleeding, major bleeding, or all-cause mortality. The pooled analyses are detailed in Supplementary Material 1, Figures S5 and S6.

In the subgroup analysis excluding conference abstracts,^8,11,21,27,29,31,34,36,37,40,44,45,47,48,50,51^any bleeding was significantly reduced in the group treated with DOACs. However, there was no significant difference between groups regarding SEE events, stroke, SE events, thrombus resolution, clinically relevant bleeding, minor bleeding, major bleeding, or all-cause mortality. The pooled analyses are detailed in Supplementary Material 1, Figures S7, S8, and S9.

### Sensitivity analysis

We performed a leave-one-out sensitivity analysis for all outcomes. There was a significant decrease in SSE events in favor of DOAC therapy omitting Robinson et al.^11^ There was a significant difference in favor of DOAC therapy in any bleeding omitting Al-abcha et al., Albabtain et al., Jaidka et al., Yunis et al., or Zhang et al. (2022).^21,23,39,46,48^ There was a significant difference in clinically relevant bleeding in favor of DOAC therapy omitting Jaidka et al., Mihm et al., or Seiler et al.^39,43,50^ There was a significant difference in thrombus resolution in favor of DOAC therapy omitting Robinson et al.^11^ The leave-one-out sensitivity analysis plots are detailed in Supplementary Material 1, Figures S10 to S18.

### Quality and evidence assessment

Individual RCT appraisals according to the RoB-2 tool are illustrated in Supplementary Material 1, Figure S19. Overall, all RCTs raised some concerns due to deviations from intended interventions,^8,49,51^ and one RCT raised some concerns due to selection of reported results.^49^

No significant publication bias was detected for the SSE outcome by Egger’s test (p = 0.702) or Begg’s test (p = 0.327). The funnel plot of the SSE outcome is available in Supplementary Material 1, Figure S21.

Critical appraisal of the cohort studies is detailed in Supplementary Material 1, Figure S20. Four cohort studies showed a low risk of bias,^29,40,47,48^ while 10 cohort studies had a moderate risk of bias, due to biases in participant selection.^11,21,27,31,34,36,37,44,45,50^ One RCT and 15 cohort studies did not provide enough information to assess the risk of bias.^22-26,28,30,32,33,35,38,39,41-43,46^

According to the GRADE assessment, very low quality was assigned to all outcomes, mostly due to the inclusion of abstracts and multiple studies with no information regarding risk of bias. Supplementary Material 2 reports the full GRADE assessment and summary of findings.

### Trial sequential analysis

The cumulative z curve for stroke and clinically relevant bleeding did not surpass the conventional and monitoring boundaries and did not reach the required information size (RIS). In this case, we cannot conclude whether the neutral results arise from a lack of power or the intervention is unlikely to provide a significant impact. For thrombus resolution, the last point in the z curve lies within the futility boundaries, indicating that it will unlikely reach statistical significance, even if we proceeded to include trials randomizing patients until the RIS of 367. The trial sequential graphs are detailed in Figure 6 and Figure 7.

**Figure 6:** – Trial sequential analysis for (A) stroke and (B) clinically relevant bleeding. DOACs: direct oral anticoagulants; VKAs: vitamin K antagonists

**Figure 7:** – Trial sequential analysis for thrombus resolution. DOACs: direct oral anticoagulants; VKAs: vitamin K antagonists.

## Discussion

In this systematic review and meta-analysis comprising 33 studies and 4,450 patients, we compared the efficacy of two types of anticoagulants, DOACs and VKAs, for the treatment of LVT. Our findings were: (I) DOAC therapy was equivalent to VKAs for LVT in the occurrence of thromboembolic events and thrombus resolution; (II) rivaroxaban significantly reduced thromboembolic events; and (III) the occurrence of bleeding complications was similar between groups.

LVT constitutes a prominent etiological factor for embolic stroke subsequent to AMI and congestive heart failure.^52,53^ Maniwa et al. revealed that individuals with LVT can experience SE events with an incidence rate as high as 16.3%, which is 5 times greater than those without LVT,^54^ and more than 10% die within 1 year.^55^ European and American guidelines recommend anticoagulant therapy for 3 to 6 months in patients with LVT.^6,56^ VKAs, mainly warfarin, are indicated as first-line oral anticoagulants for the treatment of LVT.^9^ Nevertheless, the use of warfarin comes with drawbacks, including interactions with drugs and food, variability in individual responses, the requirement for frequent monitoring, and the necessity of using unfractionated heparin or low molecular weight heparin for at least the 3 initial days due to a delay in factor II inhibition.^57^

In the last decade, new DOACs have been approved for anticoagulant treatment of nonvalvular atrial fibrillation (AF) and venous thromboembolic diseases.^58,59^ The anticoagulant action of DOACs is based on thrombin inhibition, with dabigatran, or factor Xa, with rivaroxaban, edoxaban, and apixaban.^57,59^ DOACS are now proven to be superior to warfarin in the treatment and prevention of thromboembolic events in patients with non-valvular AF.^60^ For LVTs, DOACs may be applicable, as the pathophysiological mechanism is similar to AF-related thrombus.^57^ However, no formal updated guideline has recommended the use of DOACs in patients with LVTs. The 2013 American College of Cardiology/American Heart Association STEMI and the 2017 European Society of Cardiology STEMI guidelines do not make reference to the use of DOACs in anticoagulation for LVT.^6,56^ Nonetheless, the 2021 American Heart Association/American Stroke Association stroke guideline featured a class IIb recommendation supporting the use of DOACs to reduce the risk of recurrent thrombosis in patients with stroke or transient ischemic attack and new LVTs.^5,9^ Thus, DOACs have been used as an off-label treatment for LVTs, with very limited guidance on their use.^57,61^

In a meta-analysis published as a scientific statement by the American Heart Association, DOACs were considered to be a reasonable alternative to VKAs in patients with LVT.^9^ This treatment approach was particularly attractive for patients for whom maintaining a consistent therapeutic INR range proves challenging or for those who cannot undergo frequent INR monitoring.^9^ In previous meta-analyses, Michael et al. showed reduced stroke rates in patients treated with DOACs compared to VKAs, with similar thrombus resolution and bleeding events.^62^ Accordingly, Trongtorsak et al. demonstrated similar rates of systemic thromboembolic events, thrombus resolution, and bleeding.^63^ Chen et al. and Kido et al. showed a significant reduction in bleeding in patients treated with DOACs compared with VKAs, with similar rates of thromboembolic events.^57,64^ In our meta-analysis, we found a significant reduction in any bleeding in favor of DOACs only in the subgroup analysis excluding conference abstracts. Considering that the exclusion of conference abstracts reduces the possibility of bias in the statistical analysis, this result favors the interpretation of the existence of a benefit of DOACs for the treatment of LVT in comparison to VKAs.

Li et al. and Ferreira et al. found no differences between DOACs and VKAs for thromboembolic events or bleeding.^61,65^ Accordingly, our meta-analysis has also indicated no statistical differences for the outcomes of stroke, SE events, or the composite outcome of SSE events. However, to the best of our knowledge, this is the first meta-analysis that performed subgroup analyses for apixaban and rivaroxaban, finding a benefit of rivaroxaban in reducing thromboembolic events, assessed by the significant reduction of SSE and SE events when compared with VKAs.

In TSA, firm evidence is reached when the patient sample size exceeds that required for achieving a definitive conclusion, or when z curves cross the TSMBs before reaching the essential patient count for conclusive evidence. Conversely, in instances in which the z curve crosses the conventional statistical thresholds but not the TSMBs and the necessary patient sample to accept or reject the hypothesis, the significant effect from the meta-analysis may stem from repetitive testing, rather than genuine underlying effects. In cases where the number of participants in the meta-analysis exceeds the RIS line, this suggests that there is sufficient evidence to draw reliable conclusions about the effect of the intervention. In our meta-analysis, TSA did not show sufficient evidence of the benefit of DOACs over VKAs for the treatment of LVT regarding thrombus resolution, stroke, and clinically relevant bleeding.

This study must be interpreted considering its limitations. First, most of the studies included in this meta-analysis were cohort studies, and the sample sizes, especially in the RCTs included, were small. Second, patient characteristics, type of DOACs, definition of clinical events, and follow-up showed variations among the included studies, contributing to inter-study heterogeneity. Third, the inclusion of conference abstracts represents a potential source of bias in the analysis, as abstracts often lack information about the patient population, and it is not possible to assess the risks of bias due to the inherent lack of detailed information about the study design. Furthermore, due to the limited information provided in the conference abstracts, population overlap is a concern, although no abstract included in our meta-analysis had the same number of patients as fully published studies. Fourth, GRADE assessment exhibited a very low quality of evidence due to significant inter-study heterogeneity and a severe risk of bias. Fifth, subgroup analyses were performed with a small sample size. Sixth, subgroup analyses for different DOACs, except rivaroxaban and apixaban, were not feasible due to a lack of adequate data. Therefore, the generalizability of the findings is limited. Larger RCTs are needed to confirm the efficacy and safety of DOACs compared to VKAs for the treatment of LVTs.

## Conclusion

DOACs had a similar rate of thromboembolic and hemorrhagic events, as well as thrombus resolution, compared to VKAs in the treatment of LVTs. Rivaroxaban therapy had a significant reduction in thromboembolic events, compared to VKAs.
